# Exploring the intersection of atherosclerosis and Alzheimer’s disease: the role of inflammation and complement activation

**DOI:** 10.1007/s00011-025-02069-6

**Published:** 2025-07-10

**Authors:** Emilia Vataja, Giorgio Ratti, Adrian Safa, Marta Pagano, Luigia Ferrante, Seppo Meri, Karita Haapasalo

**Affiliations:** 1 Department of Bacteriology and Immunology, University of Helsinki, Biomedicum 1 Haartmaninkatu 8, 00014 Helsinki, Finland; 2 Department of Pathophysiology and Transplantation, University of Milan, Via Francesco Sforza 35, 20122 Milan, Italy; 3 Department of Neurologic Surgery, Mayo Clinic, 4500 San Pablo Rd, Jacksonville, FL 32224 USA; 4 Humanitas University, Via Rita Levi Montalcini, 4, 20072 Milan, Italy; 5 Diagnostic Center, Helsinki University Hospital, Topeliuksenkatu 32, 00290 Helsinki, Finland

**Keywords:** Complement, Inflammation, Neuroinflammation, Cardiovascular disease

## Abstract

**Background:**

Atherosclerosis (AS) and Alzheimer's disease (AD) are both multifactorial in nature and share many risk factors. Vascular dementia and AD may occur together, and a substantial proportion of AD cases also have signs of cardiovascular disease, a relationship well-established by cohort studies. The risk factors could contribute to persistent smoldering inflammation, including activation of complement at sites of endothelial injury and/or by accumulation of molecular aggregates.

**Methods:**

To examine the possible bridging points between AD and AS, we constructed a comprehensive narrative review.

**Results:**

A connecting point between AD and AS is inflammation. Contrary to prior assumptions, a significant linkage exists between systemic inflammation and neuroinflammation. Activities of complement, a key effector of innate immunity, are of special interest in the pathogenesis of both diseases.

**Conclusion:**

AS and AD share a partially overlapping array of pathophysiological mechanisms.

## Introduction

### Alzheimer’s disease and atherosclerosis—similar pathophysiology?

Alzheimer's disease (AD) and atherosclerosis (AS) are age-related inflammatory conditions with shared drivers such as distinct apolipoprotein E (ApoE) allotypes, oxidative stress, and inflammation [1, 2]. Of the shared drivers, complement of innate immunity is of interest because complement proteins are deposited in the affected tissues in AD and AS [3–5], and complement is activated context-dependently, causing both protective and detrimental effects, as will later be described. Thus, to examine the potential of complement to connect the pathogeneses of these diseases, we hereby review the relevant research about the subject to construct a concise overview of this topic.

### Alzheimer's disease (AD)

AD is a progressive neurodegenerative disorder that accounts for 60–80% of cases of dementia in individuals over 65 years of age [6]. It is a disease that currently has no definitive cure, making AD a significant social and economic burden.

Clinically, patients with AD present with a progressive decline in cognitive abilities, including memory loss, and impairments in speech, coordination, and movement, depending on the severity and stage of the disease. In most cases, symptoms support an accurate diagnosis [7].

Histologically, AD is characterized by the accumulation of deposits in the brain composed of amyloid β (Aβ) protein plaques and neurofibrillary tangles (NFTs), which can be identified using amyloid or tau positron emission tomography (PET) scans [7]. Aβ peptides play a fundamental role in AD pathogenesis. They are produced by a series of enzymatic cleavages from the amyloid precursor protein (APP). The APP-derived Aβ42 peptide, in particular, is highly prone to aggregation due to its hydrophobic amino acids in the C-terminus [8] and is associated with the neurodegenerative process observed in AD. Similarly, tau, a microtubule-binding protein essential for maintaining neuronal structure and function, becomes irregularly phosphorylated and aggregated, contributing to the formation of NFTs. Through post-mortem histopathological analysis, it is possible to confirm the diagnosis of AD by detecting these pathological changes in the brain parenchyma [7].

ApoE is a component of high-density lipoproteins (HDL) in plasma and the predominant apolipoprotein in the central nervous system (CNS). Of the three ApoE isoforms, ApoE4 is the most important genetic risk factor for late-onset AD, while ApoE2 is protective [9]. Other important and strong risk factors for AD include complement genes (clusterin (CLU), complement receptor 1 (CR1)), which highlight the role of the central and peripheral complement system in the disease pathogenesis [10].

### Atherosclerosis (AS)

Cardiovascular diseases (CVDs) are the leading cause of death worldwide. In 2023, in the Western world, coronary heart disease killed over 370,000 people [11]. One of the most significant CVDs is AS, which is a chronic inflammatory disease that primarily affects the walls of large and medium-sized arteries [12]. The plaques formed in AS consist of fatty substances, cholesterol, cellular waste products, calcium, fibrin, extracellular matrix (ECM) components, and inflammatory cells that accumulate in the inner lining of arteries, known as the intima [12].

The pathogenesis of AS begins with the accumulation of lipid-rich deposits composed of low-density lipoproteins (LDL) that start to gather at sites of blood flow turbulence (bends and branches of arteries), accompanied by endothelial cell dysfunction [13]. The infiltrated LDL particles become oxidized (oxLDL) and proteolytically modified, which promotes their uptake by macrophages that eventually become cholesterol-laden foam cells [13]. The uptake of oxLDL by scavenger receptors and Toll-like receptors (TLR4 and TLR2) triggers cell apoptosis and pro-inflammatory signaling [14]. Several cytokines and growth factors secreted by the endothelium and macrophages lead to an increased production of smooth muscle cells and extracellular matrix components that promote the generation of a fibrous cap [15]. Additionally, positive feedback loops are created, as the activation of the endothelium and inflammatory cells leads to further progression of inflammation and the formation of oxLDL [16].

HDL has an important role in mitigating the risk of AS. It removes cholesterol from the periphery to the liver, promotes macrophage cholesterol efflux, and has beneficial anti-inflammatory and anti-oxidative functions. The reduced capacity of HDL to promote cholesterol efflux from donor cells correlates with atherosclerotic burden in the coronary and carotid arteries [17].

## Methods

PubMed was searched with the keywords (atherosclerosis OR peripheral artery disease OR vascular inflammation OR Alzheimer's disease OR neuroinflammation OR neurodegeneration) AND (complement OR classical pathway OR alternative pathway OR lectin pathway) AND (C1 OR C1q OR CR1 OR C3 OR C3a OR C3b OR iC3b OR CR2 OR CR3 OR C4 OR CR4 OR C5 OR C5a OR C5aR OR C5aR1 OR C5aR2 OR C7 OR MAC OR Mac-1 OR CD35 OR clusterin OR complement factor H) to find suitable articles to be included in this narrative review. Articles were chosen on the basis of a subjective evaluation of their suitability, considering the scope and extent of our review.

## Role of complement

### Complement

Complement is a central part of innate immunity, and it also contributes to the initiation of adaptive immunity [18]. It consists of more than 50 fluid-phase, membrane-bound, and intracellular proteins, and is a potent pro-inflammatory system involved in the clearance of invading microbes [15]. The complement system, with its many functions, also plays an important role in the homeostatic clearance of apoptotic cells and debris, together with phagocytes [19, 20]. Its excessive activation may, however, damage host tissues both indirectly, by recruiting and activating immune cells or stimulating endothelial and other cell types, and directly, via the cytolytic membrane attack complex (MAC, C5b-9) [21].

**Fig. 1:**
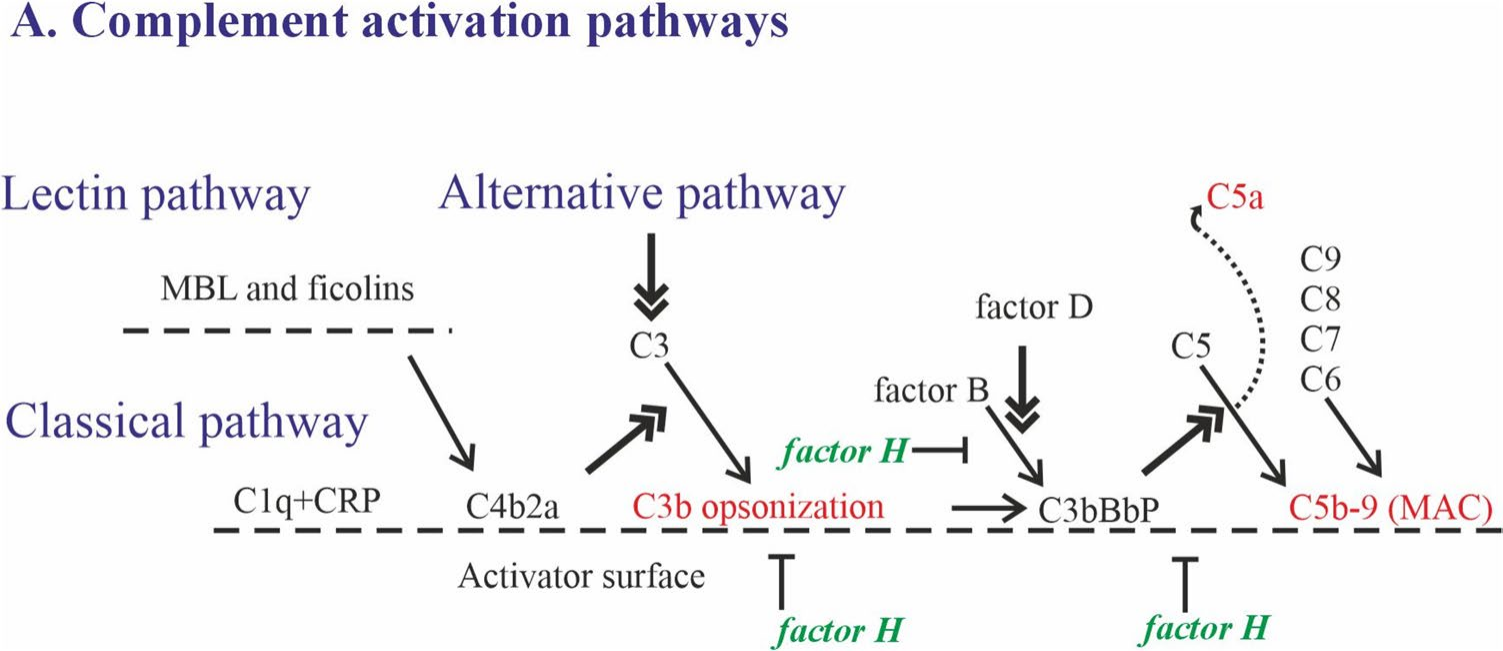
Complement activation pathways and regulation by factor H. All three pathways of the complement system (classical, lectin and alternative) converge at the level of C3 cleavage into C3a and C3b (center of the figure). Classical pathway starts from C1q binding to an antigen–antibody complex or to C-reactive protein (CRP) bound to a surface. This binding leads to the activation of C1r and C1s, the latter cleaves C4 and C2 into C4a and C4b and C2a and C2b, respectively. After binding to a surface C4b binds C2 forming the CP C3 convertase (C4b2a complex). Activation of the lectin pathway is mediated via binding of lectins (MBL or ficolins) to carbohydrate or acetylated moieties on the surface. They form a complex with MASP1 or MASP2 (MBL-associated serine proteases) that, once activated, cleave C2 and C4 forming the classical pathway C4b2a complex. The alternative pathway is spontaneously activated through hydrolysis of C3 to C3(H_2_O) in the fluid phase or through C3b formed by C3 convertase of the classical or lectin pathway (C4b2a) or of the AP (C3bBb). In the AP C3 convertase, factor B associates with C3b and becomes cleaved by factor D to generate C3bBb, that is stabilized by properdin (P). Subsequently, the same enzyme (C3bBbP) acts as the C5 convertase to cleave C5 to C5a and C5b. C5a anaphylatoxin attracts and activates inflammatory cells, while C5b-9 forms the MAC consisting of C6, C7, C8 and several C9 complement components. The main regulator of complement at the C3 level is soluble FH, which acts as a cofactor for factor I-mediated cleavage of C3b, inhibits binding of factor B to C3b and promotes dissociation of Bb from C3bBb

Activation of complement can occur via three pathways (Fig. 1): the classical pathway (CP), the alternative pathway (AP), and the lectin pathway (LP) [15]. The CP is activated by C1q binding to antigen–antibody complexes, C-reactive protein, and many tissue structures exposed during tissue damage. The LP is triggered by mannan-binding lectin- (MBL) and ficolin-mediated recognition of specific carbohydrates or acetylated microbial structures. The AP is spontaneously activated at a low level on any biological surface, and activation becomes amplified if the surface lacks the ability to downregulate complement [15]. All pathways converge at the cleavage of C3 to C3a and C3b catalyzed by the C3 convertases, eventually leading to the formation of the membrane attack complex (MAC) [21]. MAC formation is initiated by the generation of C5b, and continued by sequential deposition of components C6, C7, C8, and multiple copies of C9 to form a polymeric pore [15]. The anaphylatoxins C3a and C5a provoke inflammation by attracting and activating leukocytes via C3aR and C5aR1 [15]. MAC formation can cause activation or lysis of the target cells, while C1q, C4b, C3b, and its inactivation product, iC3b, opsonize targets for phagocytosis [20].

Due to its rapid and potentially explosive activation, complement activity is tightly regulated. Complement factor H (FH) controls amplification of the AP in the fluid phase and identifies self-surfaces with its C-terminus and regulates complement with its N-terminus [1]. Additional complement regulators with functions partially overlapping those of FH are present on all cell surfaces. These include CR1 (CD35), CD46 (membrane cofactor protein, MCP), and CD55 (decay-accelerating factor, DAF). The terminal pathway is inhibited by soluble CLU (ApoJ) and vitronectin, as well as by an important membrane regulator, CD59 (protectin) [1].

#### Complement C3 and complement receptor 3 (CR3)

The levels of the C3 protein in the CSF in AD patients are higher than in healthy controls [22, 23]. Similarly, the plasma levels of C3 in AD are higher than in the cognitively normal population both cross-sectionally [24, 25] and longitudinally [26]. Similarly, in AS, an elevated serum level of C3 predicts myocardial infarction (MI), especially in men [27], more extensive arterial calcification in middle-aged women [28], a more atherogenic plasma lipoprotein profile [29], larger carotid intima media thickness and the presence of carotid AS in rheumatoid arthritis [30], more severe renal arteriosclerosis [31], and coronary heart disease in heavy smokers [32]. Additionally, C3b and iC3b are deposited in atherosclerotic arteries, as shown by immunohistological analysis of surgically resected human atheromas [33]. However, in contrast to positive correlations with the conditions, it has been shown that low plasma C3 is associated with an increased risk for AD, especially in ApoE4 carriers [34].

To explain the association of high C3 levels in plasma, brain parenchyma, and CSF with AD and AS, multiple mouse studies have examined the effects of C3 and its activation products and CR3 or C3aR by breeding transgenic or knock-out mice or pharmacologically blocking the corresponding receptors. In an AD mouse model, neuronal overproduction of Aβ activates the release of C3 by astroglia. Upon complement activation, the C3 cleavage fragment C3a interacts with microglial C3aR to modulate microglial phagocytosis of Aβ [35]. When the Aβ-induced C3a-C3aR interaction persists, microglial phagocytosis of Aβ is attenuated, and consequently, Aβ accumulation is accelerated [35]. In line with this, C3-deficiency in mice has been shown to protect synapses from Aβ [36–38], and inhibiting or antagonizing C3, CR3, or C3aR prevents synaptic loss and dysfunction [35, 36, 39–41]. This neuroprotective effect of blocking the effect of C3 activation products on their receptors could be explained by lower levels of pro-inflammatory cytokines, less loss of neurons and synapses in the hippocampus [37], and decreased phosphorylation or amount of tau [39–41]. Some of these protective mechanisms depend on the presence of the cleavage product of C3b, iC3b, the formation of which requires CR1 or FH [42]. Therefore, it is not clear whether the loss of C3 or downstream activation of complement (iC3b-CR3 and C3a-C3aR) is of more importance in the observed neuroprotective effects. Moreover, in AD, C3a and C3aR have been implicated in the disruption of the integrity of the blood–brain barrier (BBB) because binding of C3a to C3aR on BBB endothelial cells leads to calcium-mediated loss of intercellular junction proteins [43]. While C3 and C3aR are directly related to complement activation, the role of CR3 in the loss of synapses requires formation of C3b to iC3b, which is due to irreversible cleavage of C3b by the C3b inactivator enzyme (factor I). Complement FH is known to act as a cofactor for the factor I-mediated cleavage of C3b to iC3b and thereby may contribute to the clearance of synapses labeled by iC3b. In addition, FH binds directly to the CR3 receptor and inhibits binding of Aβ to CR3 [2], thus potentially reducing the Aβ-dependent engulfment of synapses by microglia [36].

While increased C3 levels have been shown to contribute to the detrimental effects in AD, the molecular mechanisms affecting C3 levels in AS have been less explored. In an AS mouse model, C3b and C4b have been found to associate with collagen and elastin in the walls of arteries. This suggests C3b and C4b deposition on the walls of arteries, which could lead to increased stiffness of the vasculature seen in AS [44]. C3b deposition on arterial walls does not, however, explain the positive correlation between AS and C3 concentrations in AS. Instead, elevated C3 levels are most likely due to the chronic inflammation characteristic of the disease that induces C3 synthesis by hepatocytes. Similarly, induction of local C3 synthesis by macrophages and smooth muscle cells in the arterial wall may contribute to a feedback loop that provides more C3 for cleavage, amplifying the alternative pathway [45]. Moreover, extensive deposition of iC3b has been detected in ruptured atherosclerotic plaques but not in the unruptured atheromas. Thus, iC3b could promote the instability of the plaques [46]. Importantly, in healthy individuals, iC3b formation reflects a balance between complement activation and regulation. Low levels of iC3b formation indicate resolution of inflammation. In AS, which is a chronic inflammatory condition, this balance is disrupted, leading to excessive formation of iC3b deposits. The iC3b deposits interact with complement receptors CR3 (CD11b/CD18) and CR4 (CD11c/CD18) on macrophages, triggering phagocytosis and inflammatory responses [47].

In contrast to the pro-inflammatory effects of C3b and C3a, the interaction of C3 activation products with their receptors has been proposed to exert anti-inflammatory effects in AD and AS. In a mouse model deficient in C3, the animals exhibited more pronounced AD pathology, indicated by a bigger Aβ burden, higher Aβ42 levels, and increased amounts of pro-inflammatory microglia [48]. In AS mouse models, the atheroprotective effect of C3-deficiency is more pronounced. Here, C3-deficiency resulted in the formation of atherosclerotic lesions faster and an increase in serum triglyceride levels compared to wildtype controls [49]. Similarly, C3-deficiency in mice has been shown to lead to the formation of less mature atheromas that contain more lesional macrophages, and less collagen and SMCs compared to control mice [50]. In addition, C3aR-deficiency in mice has been shown to result in increased AS and more pronounced inflammatory responses in the lesions [51].

In AD, the mechanisms underlying C3-mediated neurotoxicity involve activation of astroglia and microglia, synaptic and neuronal death, increases in the amounts of pro-inflammatory cytokines, disruption of the BBB, and accumulation of Aβ and phosphorylation of tau. In AS, however, C3-mediated effects seem to exert more of an atheroprotective effect as shown by mice deficient in C3. This seems paradoxical since large cohort studies have shown that high C3 concentrations in plasma are associated with AS and its complications. However, high C3 concentrations may be a result of systemic inflammation and extensive synthesis of C3 from hepatocytes, while local C3 production from arterial walls likely triggers amplification of complement activation, which could be detrimental for plaque stability. On the other hand, balanced complement activation and regulation can be atheroprotective as it has an important role in mediating the cleaning of dead cells and debris.

#### Complement C5 and C5aR

In AD, high plasma C5a levels predict more advanced disease stages [52]. Similarly, increased plasma C5a is associated with subclinical AS [53] and endothelial dysfunction [54]. Experimental studies support these findings, although we know that C5a levels can vary widely, and C5a is rapidly removed from circulation. In AD mouse models, antagonizing the proinflammatory C5aR1 reduces inflammation and enhances induction of clearance pathways in microglia, preserves neuronal complexity [55], and prevents excessive neuronal damage and synaptic pruning [56]. Blocking of C5aR1 also decreases excessive activation of astroglia and microglia [57] and reduces Aβ load and the amount of dystrophic neurites [58]. In another AD mouse model, C5aR agonist was found to decrease the Aβ load partly by enhancing phagocytosis, preventing loss of synapses and neurons, and decreasing astrogliosis [59].

In AS mouse models, antagonizing C5aR results in decreased lesion size, intima-to-media ratio, and intra-plaque lipid content [60]. Furthermore, C5aR1 blocking decreases neointimal hyperplasia and inflammatory cell content [61]. However, another study showed that mice develop AS regardless of C5aR1-deficiency [62], suggesting a complex role for C5a in the disease progression. Nevertheless, overexpression of C5aR1 in AS mouse models results in accelerated atherogenesis via promoting macrophage accumulation in the lesions and increasing serum proinflammatory cytokine concentration [63]. Similarly, C5a and C5aR1 overexpression in an AD mouse model resulted in accelerated cognitive decline [64].

In post-mortem AD brain samples, C5aR1 is colocalized with Aβ plaques, NFTs, and dystrophic neurites [65]. In cell cultures and rat brain slices, C5aR1 signaling has been shown to increase apoptosis [66]. Furthermore, pruning of synapses has been linked, at least partly, to a signaling axis that consists of C5aR1, glutamate and N-methyl-D-aspartate receptor (NMDAR), and metabotropic glutamate subtype 5 receptor (mGlu5R) [67]. Furthermore, in AS, C5a acts through extra- and intracellular C5aRs of atheroma-resident macrophages, and the binding of C5a to C5aR increases interleukin-1β secretion and reactive oxygen species (ROS) production in vitro [68]. In mice, C5a associates with increased intra-plaque C5aR expression, hemorrhage, and apoptosis, which significantly contribute to subsequent plaque rupture [69].

#### Complement component C1q

In AD mouse model brains, amounts of C1q are increased, not only concomitantly with the plaques but also before insoluble Aβ protein is deposited [36, 70]. Similarly, in advanced and ruptured atherosclerotic plaques, C1q is more strongly expressed [71]. Furthermore, plasma concentrations of C1q are higher in individuals with pathological changes of AD in the CSF [72] and with mild cognitive impairment (MCI) progressing to AD during follow-up [73]. In contrast, decreased C1q plasma levels correlate with an increased risk for AS [74, 75] and might predict complications of AS [76]. However, both low and high concentrations of C1q in plasma have been shown to predict CVD and its complications longitudinally [77]. The differing results from these association studies may reflect dual roles of the classical pathway of complement: protective or detrimental. Indeed, protective and pathological mechanisms of C1q in both AD and AS have been found in animal models and cell cultures. In a mouse model of tauopathy, C1q tags excitatory synapses that have accumulated with tau, leading to microglia-mediated synaptic loss [70]. In AD mice, it has been shown that C1q is required for microglial synaptic pruning but not for the clearance of Aβ [78].

These data suggest that the deposition of C1q may have pathological consequences in AD. On the contrary, high-affinity binding of ApoE to C1q attenuates the activity of C1q both on Aβ and atherosclerotic plaques, suggesting that ApoE expression may mitigate the pathological effects of C1q both in AD and AS [79]. Importantly, the interaction between ApoE and C1q is not ApoE isofom-specific and thus does not explain the association of ApoE4 with AD. In addition, binding of C1q to ApoE has also been shown to promote complement activity [79]. In contrast and importantly, FH binds to ApoE in an isoform-specific manner. Protection against complement attack by Aβ plaques is associated with ApoE2 and ApoE3 [2, 80]. Thus, the reduced binding of FH to ApoE4 could play a major role in promoting neuroinflammation and damage to synapses.

In AS, the mechanistic insights into the functions of C1q have focused on its protective role. Mice without C1q exhibited larger atheromas [81]. Additionally, C1q enhanced phagocytosis, efferocytosis, and survival of macrophages [82], and favored the anti-inflammatory M2 macrophage phenotype [83, 84] in vitro. Similarly, in AD, C1q has been shown to exert neuroprotective effects in addition to the pathological effects described above. C1q increased the outgrowth of neurites and limited neuronal stress and inflammation in vitro [85]. All in all, C1q influences the pathogenesis of both AD and AS, although the functions of C1q differ between the conditions.

#### Complement regulators (CR1 and clusterin)

Levels of soluble CR1 in plasma are lower in individuals with MCI than in those who progress to AD during follow-up [73]. Moreover, CR1 on erythrocytes binds LDL [86] and Aβ [87] when the target is coated by the opsonins C1q, C3b, and C4b. This suggests a potential link between lipid metabolism, regulation of complement, and the pathogeneses of AD and AS. As CR1 removes complement-opsonized LDL and Aβ from circulation, it may reduce systemic inflammation and thus lower the risk for both conditions.

CLU is a multifunctional glycoprotein. One of its main functions is to regulate the terminal pathway of the complement system. Its CSF concentrations are low in early AD and increase as the condition progresses [88]. Moreover, CLU has been shown to colocalize with Aβ plaques and NFTs in post-mortem brain samples [89]. The localization of CLU may reflect its neuroprotective role, as its overexpression in an AD mouse model leads to the reduction of fibrillar Aβ and neurotoxicity and gliosis associated with Aβ deposits [90].

Similarly, in AS, CLU localizes to atheromas [91]. By being an apolipoprotein (ApoJ), CLU modulates lipid levels and lipoprotein function, thereby affecting the formation of atherosclerotic plaques. However, unlike in AD, mice lacking the CLU gene exhibit smaller atherosclerotic plaques [92], which seems unexpected because it has been shown that the atheroprotective effects of HDL are decreased in the absence of CLU [93]. However, these differences between AD and AS highlight the context-dependent effects of CLU. Common to both AD and AS are the functions of CLU in promoting clearance and alleviating inflammation.

**Fig. 2:**
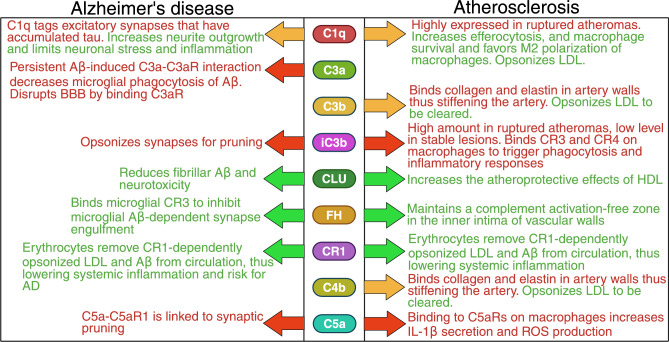
Summary of the mechanims behind the protective and detrimental effects of complement proteins in Alzheimer’s disease and atherosclerosis. Aβ = amyloid β, BBB = blood–brain barrier, CLU = clusterin, CR = complement receptor, FH = factor H, HDL = high-density lipoprotein, IL = interleukin, LDL = low-density lipoprotein, ROS = reactive oxygen species

The mechanisms of how complement proteins affect pathogeneses of AD and AS are summarized in Fig. 2.

## Complement, amyloid β, cholesterol, and apolipoprotein E

### Amyloid β, cholesterol, and complement

In AS, the significance of hypercholesterolemia as a predisposing factor for AD is widely appreciated since studies from the late 1990s already show a correlation between high cholesterol and increased risk for AD [94]. This correlation has been further supported by the observation that cholesterol-lowering medication attenuates the synthesis of Aβ and the risk of dementia [95, 96]. In mixed dementia, features common to both AS and AD typically occur.

**Fig. 3:**
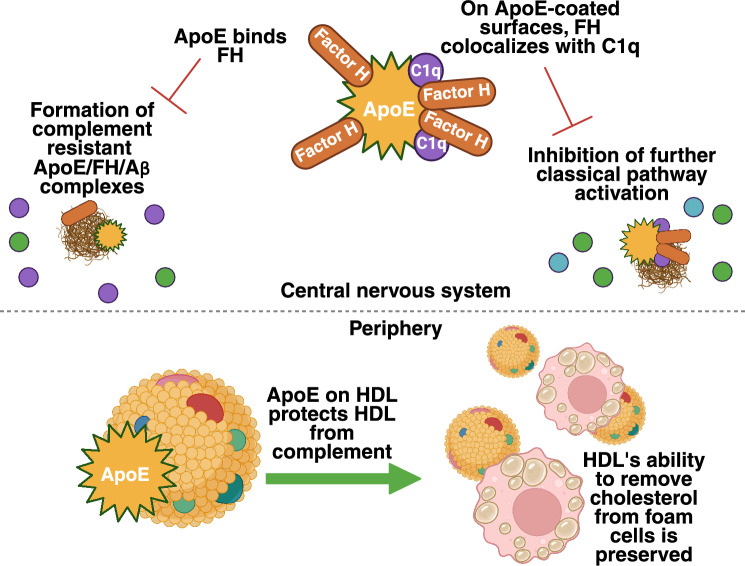
Interaction between complement, apolipoprotein E, and factor H in Alzheimer’s disease and atherosclerosis. In the central nervous system (CNS), binding of factor H (FH) to apolipoprotein E (ApoE) creates a complement-resistant complex with Amyloid β (Aβ). This leads to decreased neurotoxicity as complement cannot activate further. FH also colocalizes with C1q on ApoE. When ApoE coats a complement-activating surface, the colocalization prevents classical pathway activation. This also decreases neurotoxicity in AD. In the periphery, ApoE binds HDL to protect HDL from complement attack. This preserves the ability of HDL to remove cholesterol from atheroma-residing foam cells. Thus, this interaction works towards decreasing atherosclerotic plaque size and pro-inflammatory responses

In both AD and AS, complement proteins opsonize particulate surfaces, i.e., cholesterol crystals (CCs) and Aβ aggregates, for clearance. The alternative pathway of complement also increases the inflammatory environment through the amplification cascade, recruiting inflammatory cells and increasing the generation of inflammatory mediators. In the brain, C1q, C3, C4 [3], C1r, C5, and C9 [4] have been shown to coat Aβ plaques in post-mortem brain samples. Additionally, deposition of C1q and FH has been observed in frontal cortex samples ex vivo [2], reflecting their potential roles in the clearance of Aβ and cell debris. Furthermore, activation of C5aR1 with an agonist increases complement-mediated phagocytosis of Aβ in mice [59], suggesting that C5a contributes to the clearance pathway through the recruitment of phagocytes. In the absence of C1q and CR3, the neurotoxic effects of Aβ are diminished, as demonstrated in an AD mouse model [36]. This suggests that both C1q and CR3 regulate the neurotoxic effects of Aβ (Fig. 3).

Similarly, CCs in atheromas are coated with complement opsonins [5], as a result of activation of the CP. CCs are also opsonized by MBL and ficolin-2, activating the LP [97]. The activation of complement pathways increases the clearance of the CCs but can potentially also increase the level of inflammation via the formation of the anaphylatoxins C3a and C5a and activation of inflammasomes. Furthermore, CCs in atherosclerotic plaques can activate ECs in a complement-dependent manner, leading to the release of pro-inflammatory cytokines [98]. Similarly, in an AD mouse model deficient in C7, reintroducing C7 increased the level of interleukin 1β in the brain [99]. Lastly, complement mediates the coagulation induced by CCs by increasing tissue factor expression in monocytes, thus contributing to the formation of thrombi [100]. These findings highlight the potential of complement to exert both pathological and protective functions on opsonized surfaces in the brain and vasculature.

### Complement and apolipoprotein E

A central molecule in the control of cholesterol metabolism is ApoE, which is located on HDL particles [1]. The cholesterol metabolism control function of ApoE is most significant in the liver, blood, and brain [1]. Thus, ApoE is of interest when considering the similarities in the pathogeneses of AD and AS. Indeed, in both AD and AS, the ApoE4 allele is a predisposing factor [1].

The connection between ApoE and complement is supported by the finding that in AD, by using a statistical model, homozygotic carriership of ApoE4 and high C3 concentrations predict a heavier Aβ and tau load [101]. As described earlier, C1q binds to ApoE, thereby inhibiting or promoting further activation of the CP [79, 102], while the binding of complement regulator FH to ApoE in an isoform-specific manner limits complement activation on Aβ plaques. Importantly, FH also binds to ApoE on HDL particles, protecting HDL from complement attack. This interaction helps to sustain HDLs ability to remove cholesterol from foam cells, thereby supporting the atheroprotective function of FH. On HDL, FH binds to ApoE to confer protection against complement, but on oxLDL, FH binds to malondialdehyde (MDA)-modified epitopes, which in atherosclerotic lesions are generated as a response to inflammation [103]. Furthermore, in AS patients, the increased levels of FH on HDL associate with reduced levels of MDA epitopes on HDL, plaque stability, and the formation of specialised pro-resolving mediators (SPMs), suggesting a fundamental role for complement regulation in protection against plaque rupture in AS patients [113].

## Problems in clearance

### Aging

Since both AD and AS are age-related inflammatory conditions, it is important to note that the increased levels of complement proteins in plasma are also affected by ageing. Peripherally, the levels of C3 and C4 have been shown to be higher in the aged population [104]. Because the level of inflammation outside the central nervous system affects the level of neuroinflammation, peripheral inflammation may contribute to the rate of neurodegeneration in aged individuals. As an example, Aβ aggregates in the brain activate the complement system, while cerebrovascular HDL decreases vascular Aβ deposition and inflammation [105]. Similarly, the inflammatory environment, promoted e.g. by smoking, could promote the accelerated development of AS. Furthermore, in the brain, the level of C1q expression is up to 300 times higher in aged individuals, and its secretion from especially microglia and some neurons is accelerated [106]. Thus, in AD, aging increases the detrimental complement activation in the brain alongside the increased peripheral activation of complement. It may be that during aging, the expression of inflammatory genes is upregulated, and the clearance of complement-activating structures is decreased. Thus, the level of inflammation is increased.

### Genes

Polymorphisms in the CR1 gene [107] and the ApoE4 isoform of ApoE [1] are known to predispose to both AD and AS. Firstly, the most studied risk factor for AD is ApoE. It is expressed as three major isoforms (ApoE2, ApoE3, ApoE4), from which the ApoE4 allele increases the risk for both AS and late-onset AD [1, 2]. Interestingly, in an AD mouse model, ApoE4 up-regulated complement expression in the bloodstream [108]. As mentioned earlier, in the brain, binding of FH by ApoE2 or ApoE3 decreases the oligomerization of Ab by forming a complement-resistant complex, which decreases the toxicity of Ab and its detrimental effects in the brain [2]. However, the isoform ApoE4 binds FH with a smaller affinity, and thus, the beneficial effects of this interaction are diminished [2]. Moreover, the decreased binding of FH to ApoE4 gives space for C1q to bind ApoE4 and subsequently, activate the CP and increase neuroinflammation [2]. Importantly, the binding of FH to ApoE on Aβ reduces Aβ oligomerization. The effect of the *APOE* genotype on the risk of AD is thought to be Aβ-mediated by its effects on Aβ, as the Aβ aggregation and plaque formation rates are ApoE isoform-dependent (ApoE4 > ApoE3 > ApoE2). Thus, isoform-dependent binding of FH to ApoE may help to explain the underlying effect of ApoE isoforms on the amyloidogenic pathways.

In AS, binding of ApoE to macrophages is increased in the presence of FH [109]. This binding limits the activation of AP [109], which may be a mechanism for the ApoE isoform-dependent risk for AS. Moreover, FH V62I polymorphism correlates with the serum levels of matrix metalloproteinase 8 (MMP-8), which is a pro-inflammatory enzyme linked to cardiovascular diseases, thus suggesting an additional AD protective role for FH [110]. Thus, polymorphisms in both ApoE4 and FH may modulate the risks for both diseases.

In AD, CR1 can be divided into a fast and a slow isoform (CR1-F and CR1-S, respectively). CR1-S could increase the risk for AD by increasing the activation of complement, since its expression in post-mortem samples was decreased compared to CR1-F [111]. Furthermore, erythrocytes express CR1 for the clearance of, e.g., atherogenic lipoproteins [86] and Aβ [112]. This is of importance due to the potential of circulating Aβ to activate pro-inflammatory complement cascades. Thus, it may exacerbate the advancement of AD and AS. Furthermore, risks for both diseases are decreased when the serum LDL and very low-density lipoproteins (VLDL) levels are low since, as mentioned earlier, hypercholesterolemia predisposes to both diseases [94].

### Complement as a pharmacological target

As previously described, it has been shown in mice that the impairment of Aβ phagocytosis could be due to increased expression of astroglial C3, which further decreases microglial C3aR-mediated phagocytosis of Aβ. With a C3aR antagonist treatment in mice, the burden of plaque-forming Aβ42 was decreased, and the amount of Aβ40 concomitantly increased [35], reflecting the possibly beneficial role of blocking the C3aR. Similarly, in AD and tauopathy mouse models, antagonizing the C3aR resulted in reduced accumulation of tau in the brain and consequently rescued synaptic function [39], and decreased phosphorylation of tau [40]. Furthermore, the pharmacological blocking of C5aR was beneficial in preventing and attenuating the pathologies in mouse models of AD [55–58] and AS [60, 61]. Because complement influences the pathogeneses early on in both AD and AS, therapies targeting complement may not be so beneficial at a later stage. Still, complement-targeting medications could be beneficial in preventing disease complications, e.g., the rupture of atherosclerotic plaques. Furthermore, it is of interest whether complement-targeting therapy could be used as an adjuvant for the prevention of amyloid-related imaging abnormalities (ARIA; hemorrhage and edema) during treatment with anti-Aβ monoclonal antibodies, since the ARIAs are thought to be partly a consequence of vascular inflammation.

## Conclusions

The shared pathophysiological mechanisms between AD and AS highlight the central role of complement in disease progression. The interplay between complement components, lipid metabolism, and genetic factors offers valuable insights into the complex nature of these diseases. Continued research into the regulation of complement activity and its interactions with other pathological processes is essential for developing effective therapeutic strategies. Understanding the dual roles of complement in protection and pathology will be crucial for harnessing its potential as a target for intervention. As we advance our knowledge of these intricate mechanisms, we move closer to developing targeted therapies that can mitigate the burden of these debilitating diseases and improve the quality of life for affected individuals.

## Data Availability

No datasets were generated or analysed during the current study.

## Abbreviations

Aβ: Amyloid β
AD: Alzheimer's disease
AP: Alternative pathway
ApoE: Apolipoprotein E
ApoJ: Apolipoprotein J
APP: Amyloid precursor protein
ARIA: Amyloid-related imaging abnormalities
AS: Atherosclerosis
BBB: Blood–brain barrier
CC: Cholesterol crystal
CD: Cluster of differentiation
CLU: Clusterin
CNS: Central nervous system
CP: Classical pathway
CR: Complement receptor
CR1-F: Complement receptor 1 fast
CR1-S: Complement receptor 1 slow
CRP: C-reactive protein
CSF: Cerebro-spinal fluid
CVD: Cardiovascular disease
ECM: Extracellular matrix
FH: Factor H
HDL: High-density lipoprotein
LDL: Low-density lipoprotein
LP: Lectin pathway
MAC: Membrane attack complex
Mac-1: Macrophage-1 antigen
MASP1/MASP2: Mannan-binding lectin serine protease 1/2
MBL: Mannose-binding lectin
MGlu5R: Metabotropic glutamate subtype 5 receptor
NFT: Neurofibrillary tangle
NMDAR: N-methyl-D-aspartate receptor
OxLDL: Oxidized low-density lipoprotein
PET: Positron emission tomography
ROS: Reactive oxygen species
SPM: Specialized pro-resolving mediators
TLR: Toll-like receptor
TNF: Tumor necrosis factor
VLDL: Very low-density lipoprotein
